# Seroprevalence and risk factors of toxoplasmosis in cattle from extensive and semi-intensive rearing systems at Zona da Mata, Minas Gerais state, Southern Brazil

**DOI:** 10.1186/1756-3305-6-191

**Published:** 2013-06-25

**Authors:** Hugo Vieira Fajardo, Sthefane D’ávila, Ronaldo Rocha Bastos, Carolina Dutra Cyrino, Michelle de Lima Detoni, João Luis Garcia, Leandro Batista das Neves, José Leonardo Nicolau, Maria Regina Reis Amendoeira

**Affiliations:** 1 Post-Graduate Course in Biological Sciences – Animal Biology and Behaviour, Federal University of Juiz de Fora, Minas Gerais, Brazil; 2 Department of Zoology, Federal University of Juiz de Fora, Minas Gerais, Brazil; 3 Departament of Statistics, Federal University of Juiz de Fora, Minas Gerais, Brazil; 4 Department of Biochemistry, Federal University of Juiz de Fora, Minas Gerais, Brazil; 5 Laboratory of Protozoology, University of the Londrina state, Paraná, Brazil; 6 Laboratory of Toxoplasmosis, Oswaldo Cruz Institute – Fiocruz, Rio de Janeiro, Brazil

**Keywords:** *Toxoplasma gondii*, Epidemiology, Indirect fluorescent antibody test

## Abstract

**Background:**

Concerning the infection of humans by *T. gondii,* limited efforts have been directed to the elucidation of the role of horizontal transmission between hosts. One of the main routes of transmission from animals to humans occurs through the ingestion of raw or insufficiently cooked meat. However, even though the detection of *T. gondii* in meat constitutes an important short-term measure, control strategies can only be accomplished by a deeper understanding of the epidemiology of toxoplasmosis. The present study aimed to investigate the seroprevalence of toxoplasmosis in cattle from Zona da Mata, Minas Gerais, Brazil, and to identify associated risk factors, through an epidemiological investigation.

**Methods:**

The animals studied (*Bos indicus,* breed Nelore or Gir) were reared in the Zona da Mata micro-region and killed at a commercial slaughterhouse at Juiz de Fora, Minas Gerais state. The animals came from 53 cattle farms with extensive (predominantly pasture feeding management) or semi-intensive (food management based on grazing, salt mineral and feed supplementation) rearing systems. Blood samples were collected from 1200 animals, and assigned to *Indirect Fluorescent Antibody Test*.

**Results:**

When analyzing IgG anti-*T.gondii* we found an overall seroprevalence of 2.68%. In Brazil prevalences vary from 1.03% to 60%. Although in the present study, the seroprevalence per animal is considered low compared to those observed in other studies, we found out that of the 53 farms analyzed, 17 (34.69%) had one or more positive cattle. It is a considerable percentage, suggesting that the infection is well distributed through the Zona da Mata region. The results of the epidemiological investigation showed that the main risk factors of *Toxoplasma gondii* infection are related to animal management and to the definive host. There was a relationship between the number of seropositive cattle and the presence and number of resident cats, presence and number of stray cats, presence of cats walking freely, rat control by using cats and feed storage.

**Conclusion:**

These results may contribute to the development of preventive strategies in Brazil and other developing countries were extensive and semi-intensive cattle rearing systems are very widespread and the efforts to control this important zoonotic disease have attained little success.

## Background

Toxoplasmosis is a widespread zoonotic disease [1,2] caused by *Toxoplasma gondii*, an obligate intracellular coccidian parasite which forms tecidual cysts and presents a facultative heteroxene life cycle, with an asexual phase in several tissues of omnivores or herbivorous intermediate hosts and a sexual phase in carnivorous hosts [3,4]. The infection caused by this parasite is very frequent in a range of avian and mammalian species, including humans [5-8].

The two main routes of horizontal transmission from animals to humans can occur through the ingestion of the oocysts in the water or food contamined by cat feces and by the ingestion of tissue cysts present in raw or insufficiently cooked meat. The absence of meat inspection rules aimed at the detection of *T. gondii* may be a critical contributing factor to the dissemination of this disease [4,9].

Concerning the infection by *T. gondii* in humans, several studies have focused on congenital toxoplasmosis [10-12]. Limited efforts have been directed to the elucidation of the role of horizontal transmission between hosts [4,13,14]. Only a small percentage of the infections by *T. gondii* are acquired through vertical transmission and the post-natal infection routes may vary to a great extent according to the ethnic origin and geographical location [4]. In this sense it is required that the realization of studies should be aimed at the identification of the main transmission routes for a given population and ultimately the establishment of disease control strategies and infection prevention [4].

Serological studies have demonstrated infection by *T. gondii* in several animal species [14-26]. Despite the great improvement of immunologic and molecular biology techniques [27-29], commonly used in detecting *T. gondii* in the laboratory, these methods were not sufficiently explored in detecting this parasite in food destined for human consumption [4,9]. Parasitic zoonotic diseases transmitted through meat ingestion cause significant economic losses and affect the health of the entire world’s population [4,9]. The efforts to control these diseases have attained little success, especially in developing countries. The visual inspection cannot be used as the control of *T. gondii*. Other direct methods are impracticable, taking into account the microscopic dimensions of the tissue cysts and the fact that they can be located in any tissue or organ, randomly distributed and with low parasitemia [9]. In this context the indirect demonstration of the parasite through immunologic diagnostic methods may be an alternative to the fast and efficient detection of *T. gondii* in animals destined for human consumption [4,9].

Even though the detection of *T. gondii* in animal meat constitutes an important short-term measure, control strategies can only be accomplished with the deeper knowledge of toxoplasmosis epidemiology. Few studies have been conducted, aiming to identify risk factors correlated to the acquisition of infection by *T. gondii* in animals destined for human consumption. The identification of risk factors is essential to the creation of adequate methods for animal rearing management that should promote disease control and prevention and ultimately, the production of meat for human consumption free of infectant forms of this parasite.

Infection by *T. gondii* in cattle was demonstrated by several authors throughout the world [6,19,20,22,24,30-34]. However, the role of these animals in the transmission of toxoplasmosis to humans remains unclear [13,14]. Brazil is the world’s second largest producer of bovine meat. Minas Gerais has one of the largest cattle herds, with approximately 21 millions heads. Although Brazil has an important role in bovine meat production, few studies have been conducted aiming to detect *T. gondii* infection in cattle [14,33,35,36]. The studies focusing on the comprehension of the epidemiology of toxoplasmosis in animals are even more scarce [37,38]. In Minas Gerais studies have been carried out by Costa and Costa [39] at Poços de Caldas and Passos *et al*. [40], in Belo Horizonte, but until now, no studies have been carried out in the Zona da Mata region.

The main aim of this study was to investigate the seroprevalence of *T. gondii* infection in cattle from Zona da Mata, Minas Gerais, using an Indirect Immunofluorescence Reaction, and to identify risk factors associated with toxoplasmosis, through an epidemiological investigation.

## Methods

### Animals and study area

The animals studied (subspecies *Bos indicus,* breed Nelore or Gir) were reared in the Zona da Mata micro-region and killed at a commercial slaughterhouse at Juiz de Fora, Minas Gerais state. We considered the animals’ sex, rearing system and management. The rearing system of farms surveyed was classified as extensive (predominantly pasture feeding management) and semi-intensive (food management based on grazing, salt mineral and feed supplementation). At all of the farms surveyed the cattle do not have access to any type of animal origin food. Blood samples were randomly collected and all animals constituted one study group. The sample size was determined through the software Epi-Info, considering an error of 5% and an infection frequency of 50% in the bovine population from Brazil. The size of the bovine population from Zona da Mata micro-region was obtained at IBGE website, census for 2007.

### Sampling

The blood collection was conducted from January to December 2009, at a commercial slaughterhouse under the federal inspection service, located at the Municipality of Juiz de Fora, Minas Gerais. Once a month, one hundred blood samples were collected in 12 ml glass tubes, without anti coagulant, totalling 1200 samples, from 1200 animals. The tubes were labelled and transported to the laboratory in thermic bags at 4 to 8°C. The serum samples were obtained through centrifugation at 3,200 rpm during 5 minutes and stored in triplicate at −20°C.

### Indirect Fluorescent Antibody Test (IFAT)

The antigen was prepared with formolized *T. gondii* tachyzoites obtained from infected mice. The tachyzoite concentration was adjusted to 1x10^7^ per ml of phosphate-buffered saline (PBS-0.1 M phosphate, 0.33 M NaCl, pH 7.2). 10 μl aliquots of antigen were added to each well on the slides. The slides were dried at 37°C for the adhesion of the antigen. To obtain the real titer of IgG antibody, the sera were serially diluted 1:64; 1:256 and 1: 4096 from an initial dilution of 1:16. Ten microliters of each diluted serum sample was placed on the well of the slides and incubated in a humidified chamber at 37°C for 30 minutes. Slides were washed in PBS (three times, 10 min), dried and incubated for 30 min at 37°C using Rabbit anti-bovine IgG conjugate (SIGMA®, Whole molecule - diluted in PBS as 1:800). The slides were washed and counterstained with 1% Evans Blue. Finally, the samples were observed under an immunofluorescent microscopy (Nikon-Labophot-2, objective E PLAN 40× magnification and ocular lens CFWE 10xA/18).

### Epidemiological investigation

The animals analysed in the present study came from 53 cattle farms. These farms presented on average 212.32 ± 189.96 animals (minimum: 30, maximum: 1200).

A questionnaire was designed to determine the risk factors associated with toxoplasmosis and included information on ^(1)^farm size and number of animals; ^(2)^rearing system and food offered; ^(3)^feed storage facilities; ^(4)^main sources of water; ^(5)^vaccination and deworming; ^(6)^frequency of the purchase and sale of animals; ^(7)^quarantine regime for the recently purchased animal; ^(8)^veterinary examination prior to purchase; ^(9)^veterinarian attendance on the farm; ^(10)^knowledge about the disease; ^(11)^main clinical signs and most reported complaints; ^(12)^ abortion and stillbirth frequency; ^(13)^visits by public inspection agency representatives; ^(14)^rat control on the farm; ^(15)^presence of resident cats, stray cats and other potential host animals, was provided to each farm. We visited each farm and interviewed the farm owner or the employee responsible for the animals’ management. We identified questionnaire respondents in 49 farms of the 53 visited. The purpose of this questionnaire was to determine the risk factors associated with toxoplasmosis. All questionnaire respondents signed an Informed Consent Form, which provided a clear description of the objective of the study. During the visits the installation conditions were observed. Additional information about the animals, like sex and weight were taken at the slaughterhouse, during sample collection. The serological results were analysed considering the epidemiological data. The present study was approved by the ethics committee for human research of the Juiz de Fora Federal University (protocol 244/2010).

### Criterion for exclusion of animals

We excluded animals from the study when cattle owners had not authorized the use of information concerning their farms or animals.

### Statistical analysis

The data was analysed through chi square and Kruskall-wallis tests, with a confidence interval of 95%*,* with the Biostat 5.0 softwer and IBM SPSS Statistics 19.

## Results

### Prevalence

Among the 1195 animals tested for IgG anti-*T. gondii*, using the IFAT, 32 showed seropositivity, corresponding to the prevalence of 2.68%.

We observed the greatest percentage of infected animals (7.29%) in August and the lowest one (0%) in July. We found two titres of anti-*Toxoplasma gondii* antibodies, 1:64 (2.09%) and 1:256 (0.58%). Among the 53 farms analysed, 17 (32.07%) showed one or more positive animals. Among the 49 farms with questionnaire respondents, 17 (34.69%) showed one or more positive animals. We considered these farms as “positive for *T. gondii* infection”. We considered the remaining farms as “negative”, whose animals tested were not reactive in the immunological tests, even though we did not test the entire cattle herd from such locations. Among the 1195 tested animals, 736 were males and 459 were females. The percentage of infected males (1.76%) was higher than that of infected females (0.92%). However, there was no significant difference between the average number of infected males and females (p = 0.6009; H = 0.2737).

### Farm Size and number of animals

The farms analyzed had an average size of 355.97 ± 322.08 ha (30 to 1450 ha). There was no significant difference in the average values of size and number of matrices among the farms with the presence of positive animals and those without *T. gondii* positive animals.

### Management of animals

#### Rearing system and food offered

It was observed that 35 (71.42%) of the farms adopted the extensive system. There was no significant difference in the number of farms with positive animals, when comparing the two rearing systems (p = 0.1752 *χ*^2^ = 0.1546). However, the average number of infected animals from the farms with extensive rearing systems (1.2 ± 0.42; 1–2 infected animals; n = 285 examined animals) was significantly lower when compared to farms with a semi-intensive system (2.85 ± 1:34 1 to 5 infected animals; n = 257 examined animals) (H = 8.27; p = 0.0040). There was no difference in the average prevalence of infected animals from farms with an extensive rearing system and semi-intensive.

#### Feed storage facilities and main sources of water

The *χ*^2^ test (p = 0.010) showed that farms with a semi-intensive system more frequently stored feed, compared to the farms with an extensive system. All of the farms with a semi-intensive system stored feed, while only 40% of the farms with an extensive system do. There was a significant difference (p = 0.046; *χ*^2^ = 3.971) in the number of seropositive animals among the farms that store feed (26 positive animals) and those who do not store feed (6 positive animals). There was no significant difference in the number of *T. gondii* positive animals in the farms that have, or do not have at least one water source.

### Vaccination and deworming

The data related to animal health revealed that 100% of the farms comply with the vaccination schedule proposed by the Brazilian National Council of Beef Cattle, which covers the main diseases such as rabies, FMD, Brucellosis, Tuberculosis and Clostridiosis among other diseases. Already with regard to deworming, less than half of the farms (40.81%) are properly controlled. There was no significant difference between IgG anti-*T.gondii* positive and negative animals associated with the correct worm control (p = 0.0616, *χ*^2^ =3.4942).

### Frequency of purchase and sale of animals and quarantine regime

Analyzing the frequency of animal purchases, it was observed that among the 49 surveyed farms, on average 19.08 ± 22.21 animals per year are sold, with a minimum of 0 (no animals) and a maximum of 100 animals. There was no significant difference in the number of infected animals between the farms that sold above or below the average (*χ*^2^ = 0.1329; p = 0.7154). There was no significant difference in the number of animals purchased by positive and negative farms (H = 0.0090 and p = 0.9243). Among the 49 farms analyzed, 43 (87.75%) did not submit the recently purchased animal to a quarantine regime. There was no significant difference in the number of positive and negative animals among the farms that submit newly acquired animals or not, to quarantine (*χ*^2^ = 2.9864 and p = 0.084).

### Veterinary attendance on the farm, veterinary examination prior to purchase

Slightly more than half of the farms (51.02%) asked a veterinarian to examine the animals prior to purchase. There was a significant difference in the number of *T. gondii* positive animals among the farms in which clinical examinations were performed (12 positive animals; prevalence 1.03%) and in those which clinical examinations were not performed (20 positive animals; prevalence 1.73%) prior to purchase (*χ*^2^ = 8.221, p = 0.004). There was no significant difference in the number of positive and negative animals between farms with different regimes of frequency of veterinarian visits (*χ*^2^ = 10.567, p = 0.159). However, when analyzing a specific category within this variable, “only when a problem arises,” the adjusted standardized residuals in the calculation of the chi-square test were greater than 1.96 (2.5), indicating significant association between this category and the number of seropositive animals.

### Knowledge about the disease and visits by public inspection agency representatives

Among the respondents of 49 farms surveyed, 33 (67.34%) have never heard of the disease and do not know if the cattle can transmit toxoplasmosis. There was no significant difference in the number of *T. gondii* positive animals among the farms in which respondents said they have or have not heard of toxoplasmosis (*χ*^2^ = 2.194; p = 0.139) and between the farms in which the respondents claim to know whether or not the cattle can transmit the disease (*χ*^2^ = 2.537 and p = 0.111).

There was no significant difference in the number of *T. gondii* positive animals between farms with greater or lesser frequency of visits by specialized government agency representatives.

### Main clinical signs, most reported complaints, abortion and stillbirth frequency

The number of *T. gondii* positive animals from farms that have a history of abortion or stillbirth was significantly higher (32, 100% of the positive animals) when compared to animals from farms without a history (*χ*^2^ = 7.935; p = 0.019). A larger number of *T. gondii* positive animals were also observed in the farms with a history of animals with neurological disorders (n = 28, 87.5% of the positive animals) (*χ*^2^ = 15.273; p < 0.0001). The main animal health problems, reported by the questionnaire respondents were ticks (22.6%); accidents (16%); apathy (14.6%); intoxication (10.6%); gastrointestinal disorders (14.6%); sudden death (6.6%); *Carbunculus* (4%)*;* natural death (4%); tick disease (2.6%); rabies (1.3%); pneumonia (1.3%) and skin damage (1.3%).

Separately analyzing each clinical complaint showed no significant relationship with the seropositivity (*χ*^2^ = 17.6855; p = 0.0605). However, the intoxication as a specific category was significantly associated with seropositivity (*χ*^2^ = 42.521; p = 0.004).

### Rat control on the farm

A highly significant difference (χ 2 = 33.4543 p < 0.0001) was observed in the number of *T. gondii* positive animals among the properties that use cats for rat control (n = 239; 20 positive animals; prevalence 1.73%) and those that do not use them (n = 885; 12 positive 1 animals; prevalence 1.03%).

### Presence of resident and stray cats

Among the 49 farms, 34 (69.38%) had cats. Regarding the number of cats living at the farms, the average number of 2.83 ± 3.2 cats per farm was observed. The average number of cats at the farms with sero-positive animals (4.64 ± 4.5 cats per farm) was significantly higher when compared to that of the farms without positive animals (1.06 ± 4.1 cats per farm) (H = 16.77, p = 0.0000). Among the 17 farms with *T. gondii* positive animals, 15 (88.23%) had cats. There was a positive correlation between the number of cats at the farm and the number of infected cattle (Spearmann t = 2.95; p = 0.009). There was no significant difference between the average number of infected animals at the farms with zero to four cats and those with more than five cats. There was no significant difference between the average number of cats at farms with an extensive system (3.1 ± 2:51 cats per farm) and those with semi-intensive rearing (6.85 ± 5.92 cats per farm). Among the 32 farms without *T. gondii* seropositive animals, 19 (59.37%) had cats.

It was observed that the 49 farms analyzed had an average amount of 1.12 ± 1.34 (0–6) stray cats. The number of farms with an above average number of stray cats showed a greater number of infected animals, compared to those with a lower number of stray cats (p = 0.0014, *χ*^2^ = 10.2265). The farms with *T. gondii* positive cattle had cats in the neighborhood more often, compared to farms without infected animals (*χ*^2^ = 105.464, p = 0.005).

### Presence of other potential host animals at the farm

Analyzing the number of species, potential intermediate hosts except cattle, the average value of 2.85 ± 0.87 species was found, with a minimum of one and a maximum of five species, among them horses, dogs, chickens, birds, goats, sheep and rabbits. The analysis encompasses both positive and negative farms. There were significant differences in the number of positive animals in relation to the variable amount of different species (p = 0.002, *χ*^2^ =16.465). As the number of species present at the farm increased, it also increased the chance of finding one seropositive bovine.

## Discussion

Several studies performed in Brazil [14,33,36,41,42] and other countries [6,20,22,24,26,34] aimed to investigate the seroprevalence of *T. gondii* in cattle, the main species consumed by humans around the world. In 2009, the effective national cattle attained more than 205 million heads, placing Brazil as the second largest producer and largest exporter of beef in the world (http://www.ibge.gov.br). When analyzing IgG anti-*T.gondii* in cattle in the Zona da Mata, Minas Gerais, Brazil, overall seroprevalence was 2.68% in tested animals. In Brazil, according to the retrospective study carried out by Fialho *et al.*[8], prevalences vary from 1.03% to 60%. Few studies have been conducted in the State of Minas Gerais. Passos *et al.*[40], using the IFA technique, found *T. gondii* prevalence in cattle of 9% in the capital Belo Horizonte. At Poços de Caldas, Costa and Costa [39], using the same technique, found 12% infection prevalence. Varaschin *et al*. [43] found a prevalence of 21.4% in goats in southern Minas Gerais.

In the present study there were two antibody titrations of anti-*Toxoplasma gondii*, 1:64 and 1:256, among the 32 positive animals. Moreover, a significant difference was found in the weight of positive and negative slaughtered animals. This result may be indicative of lower weight gain observed in infected animals confirming the acute phase of the disease. According to Dubey and Thulliez [13], 98% of cattle show titrations less than 1:1024, which is suggestive of chronic disease. Although in the present study, the seroprevalence in cattle is considered low compared to those observed in other studies, such as Daguer *et al*. [14] (41.4%), Ogawa *et al.*[41] (26.0%), Albuquerque *et al.*[33] (14.77%) and Spagnol *et al.*[42] (11.83%), we revealed that of the 53 farms analyzed, 17 (34.69%) had one or more positive cattle. It is a considerable percentage, when compared to those found by other authors, such as Marques *et al*. [44], with only 18% of the farms being positive, which suggests that the infection is well distributed through the region analysed. In the present study, the presence of antibodies in cattle proves an exposure to *T. gondii* but does not confirm that these animals are currently potencial sources of contamination to humans*.* However, the relationship found between seropositivity and the risk factors analysed, indicates that the animals may probably be exposed to reinfection due to the presence of the definitive host and the management conditions in the farms.

According to Amendoeira *et al.*[45], toxoplasmosis is asymptomatic in 90% of the cases, and can manifest in many forms with clinical signs that are easily confused with other infectious diseases. In the present study, the epidemiological questionnaire particularly focused on abortion and neurological symptoms, the most recurrent and less specific clinical signs of toxoplasmosis [43,46]. As a result, we found a relationship between these clinical signs and higher frequency of seropositive animals. In addition, a relationship was found between the questionnaire respondents reporting intoxication as a common health problem among cattle and the number of *T. gondii* positive animals. This result is reasonable, given that one of the differential diagnostics of toxoplasmosis in their neurological manifestation is the intoxication, the signs of ataxia, dizziness and unconsciousness, found in both cases [47].

There was no significant association between the size of farms and the number of seropositive animals. These results disagree with those obtained by Albuquerque *et al.*[33] and Pinheiro *et al.*[48], who observed that animals raised on farms with a size smaller than 30 ha showed higher odds of infection (60%) than those reared in farms up to 200 ha (23%) and over 200 ha (17%). Another interesting aspect of the farms studied was the number of animals. We did not find a significant difference in the total number of cattle among the positive and negative farms. This result indicates that the population size does not influence the possibility of infection, which may be related to the fact that the transmission of *T. gondii* to animals occurs mainly by ingestion of oocysts shed in cat feces, with little probability of horizontal transmission among intermediate hosts [49].

We observed two types of rearing system for which the pasture is the main food supply. According to Marana *et al.*[50] the pasture contaminated with oocysts is the main *T. gondii* transmission route for cattle and a similar risk of infection would be expected for cattle in both systems: extensive and semi-intensive. However, in this study, the average number of infected animals from farms with an extensive rearing system was significantly lower when compared to positive farms with a semi-intensive system. It was also observed that the semi-intensive farms stored feed more frequently. Also a greater number of seropositive animals were found at the farms of the two types that stored feed. This result indicates that feed storage may be a risk factor for *T. gondii* infection and it is the main factor determining the differences between the number of infected animals, observed between the extensive and semi-intensive systems. The storage of animal feed may increase the presence of rats and, consequently, cats, their predators. Cats that are potentially infected by *T. gondii* by spending time looking for rats in the feed storage locations, can defecate on the feed contaminating it with oocysts.

We also found a relationship between the number of animal species present in the farms and the number of seropositive cattle. The low specificity displayed by *T. gondii* allows that multiple species may act as intermediate hosts. According to Dubey and Lindsay [5] toxoplasmosis infection in cats is mostly determined by the rate of infection in the local avian and rodent populations. Moreover, it is possible that these animals can act as mechanical oocysts vectors as reported by several authors [51-54]. Since the cattle analyzed in this study had no access to any animal food, which could facilitate the transmission of tissue forms of *T. gondii*, it is evident that the ingestion of oocysts, shed in the feces of the definitive host is the main route of infection in cattle. As expected, there was a relationship between the number of seropositive cattle and the variables: ^(1)^presence and number of resident cats, ^(2)^presence and number of stray cats or neighboring cats, ^(3)^presence of cats walking freely on the farm, ^(4)^rat control by using cats ^(5)^feed storage. These results are according to Weigel *et al*. [55], who stated that the risk of infection by *T. gondii* is associated with the number of infected cats, because the environment becomes contaminated.

Since it is very difficult and expensive to limit access of cats to the pastures and food stores, Frenkel *et al.*[56] studied as an alternative, a vaccine against feline toxoplasmosis. The efficacy of this vaccine was 84.0%, in preventing the vaccinated cats from excreting oocysts. Similarly, Freyre *et al.*[57] using a vaccination program, observed the absence of oocysts in feces in 100% of cats analysed. Cats can eliminate millions of oocysts in feces after ingestion of bradyzoites contained in only one tissue cyst from an infected animal. These oocysts could contaminate water, food and facilities, being the main route of infection for herbivorous intermediate hosts [[58].

## Conclusion

The results of this study stand out among the main risk factors of *Toxoplasma gondii* infection in cattle, factors related to animal management at the farms and factors related to the definitive host as well. The presence of cats and the number of cats in the farms stood out as an important risk factor. These results may contribute to the development of prevention and control of this important zoonosis.

## Competing interests

The authors declare that there are no competing interests.

## Authors’ contributions

HVF and SD conceived and designed the study, performed the experiments and the epidemiological investigation, analyzed the data and drafted the manuscript; MRRA made substantial contributions to study design and critically revised the manuscript; LBN and JLN analyzed the serum samples; JLG helped in study design; RRB and CDC helped in statistical analysis; MLD helped in data collection. All authors read and approved the final version of the manuscript.
